# Human Meibum Cholesteryl and Wax Ester Variability With Age, Sex, and Meibomian Gland Dysfunction

**DOI:** 10.1167/iovs.19-26812

**Published:** 2019-05

**Authors:** Douglas Borchman, Aparna Ramasubramanian, Gary N. Foulks

**Affiliations:** Department of Ophthalmology and Visual Sciences, University of Louisville, Louisville, Kentucky, United States

**Keywords:** age, dry eye, sex, lipid, meibum, meibomian gland dysfunction, NMR

## Abstract

**Purpose:**

Relationships between tear film lipid (TFL) layer composition, structure, and function could provide insight into the etiology of dry eye. The molar ratio of cholesteryl ester (CE)/wax ester (WE) was measured in meibum from normal donors (M_n_) and compared with meibum from donors with meibomian gland dysfunction (M_MGD_).

**Methods:**

CE/WE was measured using nuclear magnetic resonance spectroscopy.

**Results:**

CE/WE was distributed into two populations with 81% distributed near 0.55 and 19% near 0.3. CE/WE were higher in donors 13 to 19 years old compared with donors 1 to 12 years old and 20 to 88 years old. CE/WE for M_MGD_ was 30% lower, 0.34 ± 0.04, compared with M_n_, 0.49 ± 0.04. There were no sex differences in CE/WE. There were no significant racial differences between the CE/WE ratios for Asians and Caucasians. The CE/WE ratio was higher for blacks and lower for Hispanics compared to Caucasians. Due to the small number sampled, confirmation of the later racial results is needed. The packing of CE and WE in the TFL layer was proposed.

**Conclusions:**

Although M_MGD_ contains much less CE than M_n_, factors other than the CE content, such as the levels of saturation and/or proteins, may be responsible for the higher order of M_MGD_. In addition to saturation, CE could contribute to the increase in order of M_n_ between 0 and 20 years of age. Observed changes in the meibum content of CE alone is not likely to influence tear film stability.

A thin layer of lipid^1^,^2^ on the tear film surface could influence the stability of tears.^3^–^8^ Most of the tear film lipid (TFL) comes from meibomian gland, but other sources such as sebaceous^9^ and lacrimal glands^10^ could contribute a minor portion of lipid to the TFL layer. Relationships between TFL layer composition, structure, and function could provide insight into the etiology of dry eye. The current study focuses on human meibum cholesteryl ester (CE) and wax ester (WE) compositional changes with age, race, sex, and meibomian gland dysfunction (MGD).

CE is a very minor component, less than 2% of most tissues, and is not miscible in phospholipid membranes.^11^–^13^ However, CE is present in significant amounts in meibum and sebum.^9^,^14^ CE became the focus of research decades ago when it was discovered that the concentration of CE increases to 50% with age in human aortic intima^15^ and is prominent in plaques that contribute to atherosclerosis.^16^,^17^

CE and WE compose most of the TFL.^4^,^12^,^18^–^27^ The CE/WE molar ratio for adults is about 0.5:1 (Table 1); it does not change with age,^28^ but it decreases with MGD.^23^ Two populations of donors have been observed, with lower and higher ratios of CE/WE.^23^,^29^ One of the earliest studies of meibum showed that CEs contain much more anteiso- and iso-branched chains compared with WEs, and the hydrocarbon chains of CE are about three times more saturated compared with WE.^18^,^22^,^26^ The hydrocarbon chains of CE are among the longest measured for lipids, up to 32 carbons in length.^18^,^30^–^33^ CE dramatically increased the phase transition temperature and decreased the phase cooperativity of WE.^28^

**Table 1:** WE and CE Composition of Human Meibum

**Reference Citation, Normal Adult Unless Indicated**	**Stearoyl/WE, mol/mol**	**Donors Sampled,** ***N***
18	0.84	1 (76 pooled)
19	0.69*	4
12	0.54	72
20	0.53	22
21	0.85	4
22	0.72	4
23	0.57	27
24	0.53*	14
25	2.7	10
26	0.46	45
Literature average, less no. 25	0.64 ± 0.05	9 Studies
Literature average, *n* > 11	0.53 ± 0.01	5 Studies
Current study, adults > 13 y	0.51 ± 0.02†	31
25, Dry eye	2.8	27
27, Dry eye	1.14	4
20, Obstructive MGD seborrheic MGD	0.50*, 0.53*	Small group
23, MGD	0.34	48

* Calculated from the weight percentage of 0.76,^39^ 1.0,^36^ 0.76, 0.72, and 0.82^35^ using a molecular weight (g/mol) of 734 for CE and 509 for WE.

† Calculated from the 1- and 0.66-ppm resonances.

Nuclear magnetic resonance (NMR) spectroscopy has been a valuable tool for the evaluation of the lipid composition in the ocular lens^34^ and human meibum lipid.^12^,^23^,^28^,^35^–^40^ The advantages and disadvantages of using NMR spectroscopy and mass spectrometry for the compositional measurement of meibum has been discussed.^40^ Confirmation of NMR resonance assignments, critical to the interpretation of the changes in the NMR spectra of meibum, has been made.^28^,^39^,^40^ The assignments for the resonances in the CH_2_ and CH_3_ region between 2.6 and 0.6 ppm allowed for the quantification of the CE/WE molar ratio in human meibum with age, sex, race, and MGD in the current study.^39^,^40^ A major advantage of the current study compared with the previous study^23^ is that in the previous study the resonance used to measure CE was dependent on the esterification of the CE. In the previous study,^23^ it was unclear if the decrease in the CE/WE ratio with MGD was due to lysis of the CE bond or a decrease in the total content of CE. In the current study, the resonances used to measure CE were not dependent on an intact CE bond. In addition, due to the stoichiometry of the cholesterol protons, the sum of the intensities of the resonances used in the current study for CE in the CH_3_ region, 2.6 to 0.6 ppm, are nine times more intense than the resonance of the CE at 4.6 ppm. In the current study, the CE/WE molar ratio of meibum was quantified from 142 human donors, 94 without dry eye, one of the largest meibum compositional studies. Because of the larger number of samples, in the current study we were more accurately able to determine the distribution of the CE/WE molar ratio and if changes in the CE/WE molar ratio occurred between the ages of 1 to 12, 13 to 19, or 20 to 68 years, as the number of samples used in the previous study^23^ verses the current study for these age groups is 14 vs. 39, 0 vs. 17, and 26 vs. 38, respectively.

## Materials and Methods

### Materials

CDCl_3_ and d-hexane were obtained from Sigma-Aldrich Corp. (St. Louis, MO, USA).

### Collection and Processing of Human Meibum

Written informed consent was obtained from all donors. Protocols and procedures were reviewed by the University of Louisville Institutional Review Board as well as the Robley Rex Veterans Affairs Institutional Review Board. All subjects were treated in accordance with the Declaration of Helsinki.

Donors were grouped into three cohorts: C_n500_, donors without dry eye who donated meibum that was analyzed previously (M_n500_) using a 500-mHz NMR; C_MGD500_, donors with MGD who donated meibum that was analyzed previously^23^ (M_MGD500_) using a 500-mHz NMR; and C_n700_, donors without dry eye who donated meibum that was analyzed using a 700-mHz NMR (M_n700_).

Collection and processing of human meibum for C_n500_ and C_MGD500_ was made on the same cohorts as published previously^23^ and discussed below.

#### Cohorts C_n500_ and C_MGD500_

Meibomian gland expression was done by compressing the eyelid between cotton-tipped applicators with strict attention to avoiding touching the eyelid margin during expression. All four eyelids were expressed, and about 1 mg of meibum was collected per individual for direct spectroscopic study. The expressate was collected with a platinum spatula and immediately spread onto the AgCl window and into 0.5 mL of tetrahydrofuran/methanol (THF/MeOH), 3:1, volume/volume, in a 9-mm microvial with a Teflon cap (Microliter Analytical Supplies, Inc., Suwanee, GA, USA). All samples were frozen under argon gas until analysis. Analyses were performed within 3 weeks of collection of the sample. Storage of the sample on AgCl windows for over 2 months under argon did not affect the sample.^23^ Prior to NMR analysis, the THF/MeOH in the microvial containing meibum rinsed from the spatula was evaporated with a stream of argon gas.

After infrared analysis and solvent evaporation, meibum was removed from the AgCl window using a series of solvents with different hydrophobicities to ensure that all lipid classes were extracted from the window. First, the AgCl window was placed with the meibum side down into a 15-mL glass scintillation vial containing 1 mL of hexane and then purged with argon gas. A glass vial rather than a plastic one was used in all protocols to avoid plasticizer contamination. The vial was sonicated in an ultrasonic bath (Branson 1510; Branson Ultrasonics, Danbury, CT, USA) for 10 minutes. The hexane was decanted into the microvial containing the meibum lipid rinsed from the spatula. The hexane was evaporated under a stream of nitrogen gas. Methanol (1.5 mL) was then added to the scintillation vial containing the AgCl window and purged with argon gas. The vial was sonicated in an ultrasonic bath (Branson Ultrasonics) for 10 minutes. The methanol was decanted into the microvial containing the meibum lipid rinsed from the spatula and was evaporated under a stream of nitrogen gas. THF/MeOH (1.5 mL) was added to the scintillation vial containing the AgCl window and purged with argon gas. The vial was sonicated in an ultrasonic bath (Branson Ultrasonics) for 10 minutes. The microvial containing the extracted meibum lipid was lyophilized for 12 hours to remove trace amounts of organic solvents. Finally, deuterated cyclohexane (0.5 mL) was added to the sample and sonicated in a bath sonicator (Branson Ultrasonics) for 10 minutes. The solution was transferred to glass NMR tubes (Sigma-Aldrich Corp.), and NMR spectra were collected. The samples never came in contact with any plastic so as to avoid plasticizers. Control CDCl_3_ spectra were run with every 11 meibum samples to ensure no impurities were present.

#### Cohort M_n700_

Meibomian glands were gently expressed by pressing the eyelid with a fingertip with strict attention to avoiding touching the eyelid margin during expression. All four eyelids were expressed, and approximately 0.5 mg of meibum lipid was collected per individual for direct spectroscopic study. The expressate was collected with a platinum spatula under a slit lamp, and the pool of infant meibum was immediately dissolved into 0.5 mL of CDCl_3_ in a 9-mm microvial with a Teflon cap (Microliter Analytical Supplies, Inc.). Argon gas was blown over the samples to prevent oxidation. The sample in the vial was capped and frozen under argon gas until analysis. Analyses were performed within 3 weeks of collection of the sample. The samples never came in contact with any plastic to avoid plasticizers. Control CDCl_3_ spectra were run with every 11 meibum samples to ensure no impurities were present.

### Clinical Diagnosis

Clinical diagnosis was made on the same cohorts as published previously^23^ and discussed below.

Subjects were recruited from the Kentucky Lion's Eye Center and the Robley Rex Veterans Affairs Medical Center in Louisville, Kentucky. Normal status was assigned when the subject's meibomian gland orifices showed no evidence of keratinization or plugging with turbid or thickened secretions and no dilated blood vessels were observed on the eyelid margin.

The diagnosis of MGD was made according to the criteria of Foulks and Bron.^41^ Plugging of the meibomian glands of at least 5 out of 10 orifices in the central portion of the upper eyelid was required for diagnosis of MGD. The secretion expressed by the meibomian gland had to be turbid, turbid with clumps, or paste-like. Inflammation of the eyelid margin, as evidenced by both swelling of the eyelid margin, and 2+ vascular engorgement of the posterior eyelid margin were necessary for diagnosis. The presence of telangiectasia of the posterior eyelid margin was confirmatory of chronic disease but not required for entry. Tear film stability was determined by instillation of sodium fluorescein into the tear film. Tear breakup time was less than 5 seconds for all subjects in C_MGD500_.

### NMR Spectral Measurements

#### Cohorts C_n500_ and C_MGD500_

Spectral data were acquired with a spectrometer (Inova-500; Varian, Lexington, MA, USA). The following parameters were used: 800 scans were acquired with a spectral width of 15 ppm, 60 degree pulse, 4K data points, 1.0-second delay time, and 2.049-second acquisition time at 25°C.

#### Cohort C_n700_

Spectral data were acquired using a 700-MHz NMR spectrometer (VNMRS; Varian) equipped with a 5-mm ^1^H{13C/^15^N} ^13^C-enhanced cold probe (Varian Inc., Palo Alto, CA, USA). Spectra were acquired with a minimum of 250 scans, 45-degree pulse width, and a relaxation delay of 1.000 second. All spectra were obtained at 25°C. The tetra methyl silane resonance was set to 0 ppm.

Commercial software (GRAMS 386; Galactic Industries Corp., Salem, NH, USA) was used for phasing, curve fitting, and integrating.

## Results

The demographics of human meibum donors are presented in Table 2.

**Table 2:** Cohort Demographics of Donors Without Dry Eye

**Cohort Without Dry Eye**	**Number**	**Average Age, y**	**CE/WE, mol/mol**	***P***
Female	27	20 ± 3	0.51 ± 0.04	Male vs. female
Male	64	23 ± 3	0.50 ± 0.03	0.85
Caucasian	72	22 ± 1	0.51 ± 0.01	Race vs. Caucasian
Asian	7	23 ± 1	0.45 ± 0.02	0.99
Black	6	24 ± 2	0.60 ± 0.02	<0.001
Hispanic	3	11 ± 3	0.40 ± 0.04	<0.001

Values ± standard error of the mean.

### Resonance 4.6 ppm From CE

**Figure 1:**
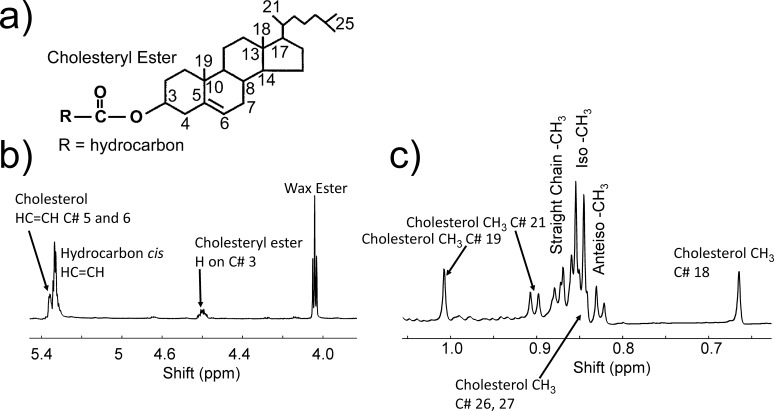
(a) Numbering used in (b) and (c) associated with CEs. (b, c) A typical NMR spectrum of meibum from a 31-year-old male Caucasian donor. The resonance due to WEs near 4.0 ppm is a triplet. The resonance due to cholesterol number C21 is a doublet. Resonances for straight chain CH_3_ and anteiso-CH_3_ moieties are composed of two major resonances, and the iso-CH_3_ moieties are composed of two major and one minor (left shoulder) resonance. The resonances due to cholesterol numbers C26 and 27 are not resolved but rather are buried under the straight chain resonances.

**Figure 2:**
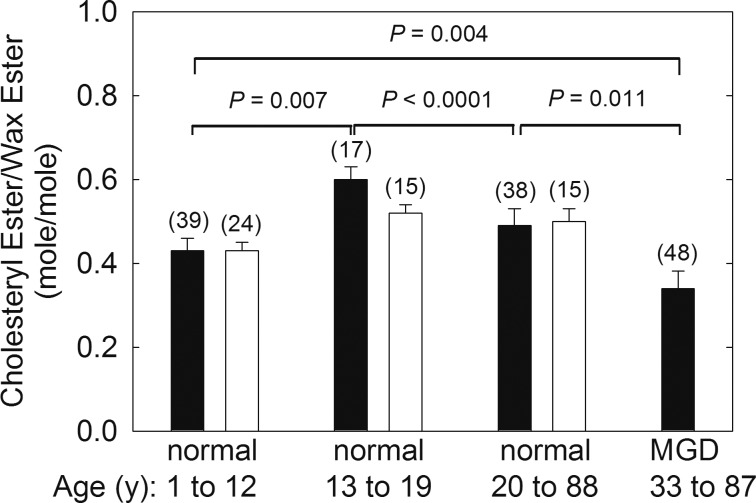
CE/WE molar ratios calculated from the NMR spectra of meibum. (Solid bar) Molar ratios calculated from the intensity of the CE resonance at 4.6 ppm and the WE resonance at 4.0 ppm. Correcting for (O)-acylated ω-hydroxy fatty acids, the solid bars would be lower. For instance, the value for MGD corrected would be 31, lower than the reported value of 34. (Open bar) Molar ratios calculated from the intensity of the CE resonances from cholesteryl numbers C18 and 19 and the WE resonance at 4.0 ppm. See Figure 1 for details.

**Figure 3:**
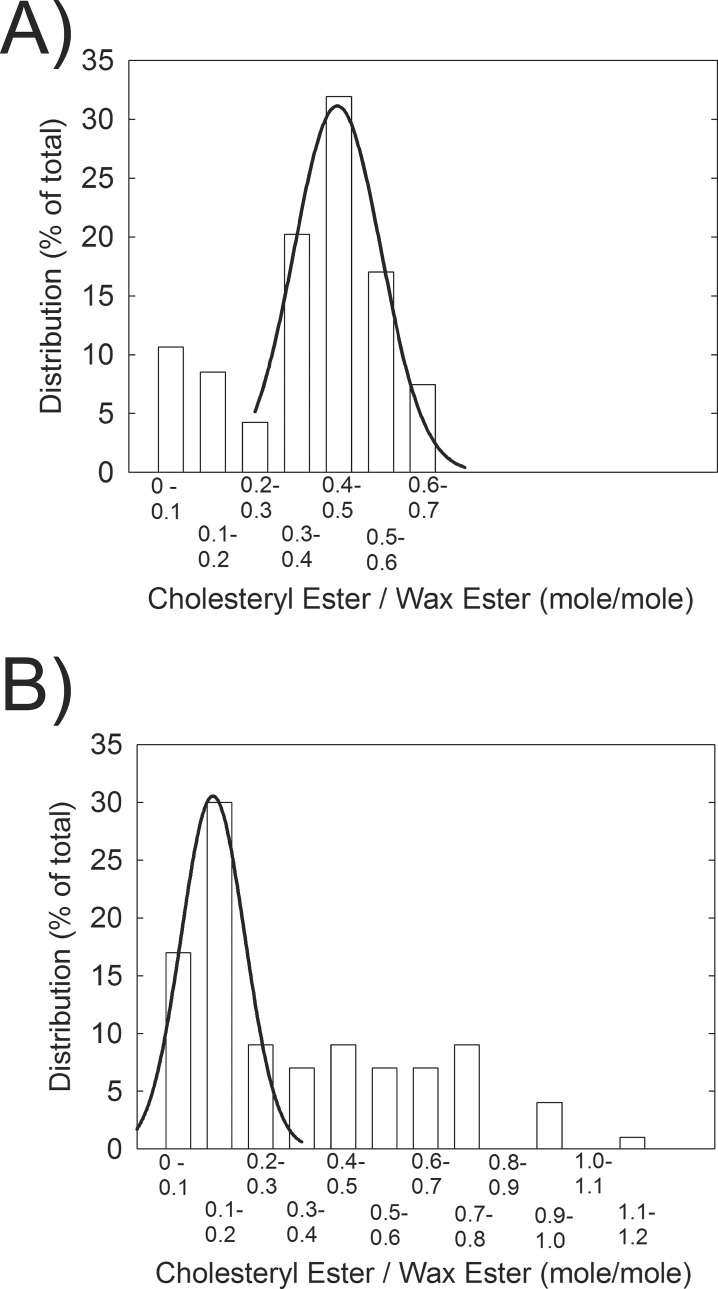
Distribution of CE/WE molar ratios calculated from the intensity of the CE resonance at 4.6 ppm and the WE resonance at 4.0 ppm. (A) Meibum from donors without dry eye. (B) Meibum for donors with dry eye.

The ^1^H-NMR spectra of all CEs, regardless of the ester hydrocarbon type, have a well-resolved broad resonance at 4.6 ppm (Fig. 1b). The ^1^H-NMR spectra of all WEs, regardless of the type of ester hydrocarbons, have a well-resolved triplet resonance near 4 ppm (Fig. 1b). From the relative intensity of these resonances, we can calculate the molar ratio of CE to WE in meibum (Fig. 2, solid bar). CE/WE was distributed into two populations, with 81% normally distributed, with a peak between 0.5 and 0.59 mol/mol, and 19% between 0.1 and 0.29 mol/mol (Fig. 3). The CE/WE molar ratios were mostly distributed in the higher ratio group for M_n_, whereas the ratios were mostly distributed in the lower ratio group for M_MGD_ (Fig. 3). The distribution was not related to age as the average age of the low and high CE/WE populations was 20.5 and 19.9 years old, respectively. The molar ratio of CE/WE were slightly but significantly higher in donors 13 to 88 years old compared with donors 1 to 12 years old and 20 to 88 years old (Fig. 2). The molar ratio of CE/WE for M_MGD500_ was significantly 30% lower, 0.34 ± 0.04, compared with M_n_, 0.49 ± 0.04 (Fig. 2). Data measured on the 500-mHz instrument was not significantly different from the data collected on the 700-mHz instrument, *P* = 0.888.

### Cholesterol CH_3_ Resonances From Carbons 18 and 19 of Cholesterol Near 0.66 and 1 ppm

The CE/WE molar ratios were also measured using the areas of the cholesterol CH_3_ resonances from carbons 18 and 19 of cholesterol near 0.66 and 1 ppm (Fig. 1a and c) and the WE resonance near 4 ppm (Fig. 1b). The molar ratios of CE/WE calculated using the 4.6-ppm resonance (Fig. 2, solid bars) and the 0.66- and 1-ppm resonances (Fig. 2, open bars) were almost identical. The intensity of the resonances at 0.66 and 1 ppm measured for M_n500_ and M_MGD500_ were low compared with those measured for M_n700_, which resulted in the intensity ratio (0.66 ppm + 1 ppm)/4.6 ppm of 1.3 for M_n500_ and M_MGD500_, not near the expected stoichiometry of 6. Thus, the data for M_n500_ and M_MGD500_ were not included in Figure 2 (open bars).

There were no differences in the CE/WE molar ratio of meibum from female donors compared with male donors (Table 2). There were no significant racial differences between the CE/WE ratios for Asians and Caucasians (Table 2). The CE/WE ratio was higher for blacks and lower for Hispanics compared to Caucasians (Table 2).

## Discussion

Relationships between TFL layer composition, structure, and function could provide insight into the etiology of dry eye.

### Meibum CE/WE Composition and Distribution

In the current meibum compositional study of 142 donors, one of the largest to date, the CE/WE molar ratio for donors without dry eye was found to be distributed into two populations, one with a peak near 0.55 and the other between 0 and 0.2. The distribution is much greater than the experimental error of approximately 5%. The distribution pattern confirms earlier studies with much smaller cohorts.^23^,^29^ The wide distribution could explain the variation in the literature values for the CE/WE molar ratio, especially for studies with fewer than 10 donors (Table 1). The CE/WE molar ratio calculated herein, 0.51 ± 0.02, was almost identical to the average literature value of studies with *n* > 11 (Table 1) and was very similar to 0.54, a NMR study from another group.^14^ The agreement between the ratios measured in the current study using different resonances for CE and the agreement with other studies confirms the reliability of the results of the current study.

There is another advantage of using the 1- and 0.66-ppm resonances to calculate CE as was done in this study and not in our previous study.^23^ The NMR data ratio from the 4.6- and 4-ppm resonances in the previous study^23^ does not account for the esters from (O)-acylated ω-hydroxy fatty acids (OAHFA). CE of OAHFA and free OAHFA account for about 3% each of the total meibum esters.^22^,^24^ OAHFA esters would add to the WE resonance near 4 ppm. CE of OAHFA would contribute one ester bond, and free OAHFA would contribute two esters to the NMR WE value. Correcting the NMR value of WE for the OAHFA esters, the corrected CE/WE ratio for M_MGD_ would be 0.31, lower than the value of 0.34 reported.

The CE/WE ratio for the age group 1 to 12 years old was statistically 28% lower than the groups older than 12 years. There were no sex differences in the CE/WE ratio. It is interesting that the CE/WE ratio was higher for blacks and lower for Hispanics compared to Caucasians. Due to the small sample sizes, even though the results were statistically significant, the results should be viewed cautiously until cohorts of at least 15 donors are analyzed. No difference was found between the CE/WE of meibum from Asians and Caucasians, but the sample size was small. One compositional study of Asians^25^ gave a CE/WE molar ratio four times higher than the average value for Caucasians (Table 1), but the accuracy of the ratio has been challenged.^24^ Therefore, further studies are needed to determine the contribution of race to the CE/WE molar ratio.

The molar ratio of CE/WE for M_MGD500_ was, significantly, 30% lower compared with M_n_. In a comprehensive study of Asians with dry eye, no difference between M_n_ and meibum from donors with dry eye was observed.^25^ However, the molar ratios in the study^25^ were very high as discussed above.^25^ In a later, smaller study by the same group,^27^ the CE/WE ratio was 59% lower with dry eye compared with M_n_,^25^ a percentage change that is in agreement with the current study. Another study using thin-layer chromatography noted a 6% decrease in the ratio of CE/WE for meibum from donors with obstructive MGD compared with M_n_.^20^

### Contribution of CE/WE to Structure

**Figure 4:**
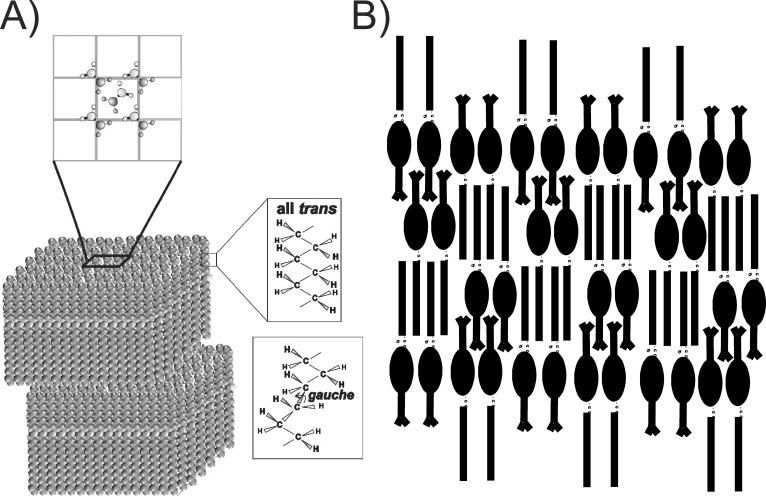
(A) Potential lamellar packing of WE. (Top) Shows rhombic packing of the hydrocarbon chains. (Right) Trans orientation for ordered hydrocarbons, gauche rotamer orientations for disordered hydrocarbon chains.^47^ (B) Smectic phase packing of CE.^17^,^61^

**Figure 5:**
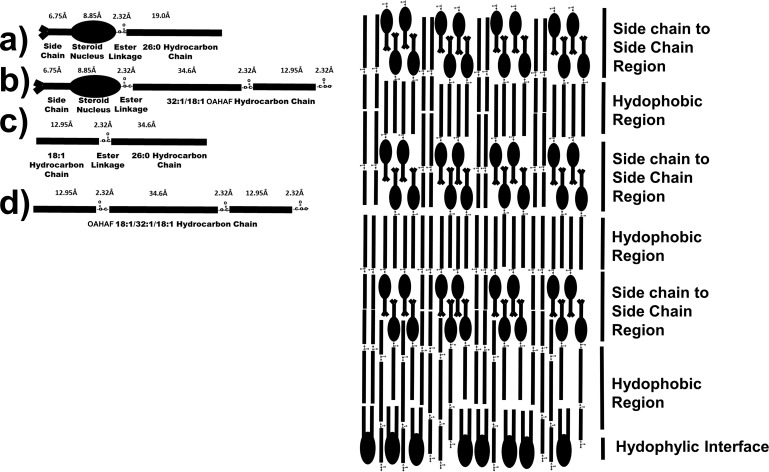
(Right) Speculative schematic of WE, CE, phospholipid, OAHFA, cholesteryl OAHFA, and OAHFA diester packing on an aqueous surface from X-ray crystallography of pure WE and CE in Figure 4 (a–d). The molecular size of the moieties were calculated from the data in Janiak et al.^12^

Infrared^9^,^42^–^54^ and fluorescence anisotropy^42^ studies show that meibum lipid hydrocarbons align to maximize Van der Waal's interactions between chains. Therefore, the WE and CE hydrocarbon chains are not randomly orientated as they are in an oil phase, as many schematic pictures in literature show them to be. At lower temperatures, meibum is in a liquid crystalline phase. The term “liquid crystalline phase” is used because meibum is not a solid crystal (100% *trans*) as the meibum hydrocarbon chains contain 72% *trans* rotamers, allowing them to pack tightly together (Fig. 4A).^54^ Thus, the term “liquid crystalline phase” is used rather than crystalline phase. At higher temperatures, meibum is in the gel phase, and the conformation of the meibum lipid hydrocarbon chains are 18% *trans* rotamers (Fig. 4A).^54^ Thus, meibum is not a liquid (0% *trans*) but rather in the gel phase. X-ray crystallography of WE (Fig. 4A) shows that WE packs together in lamellar layers with their hydrocarbons, with terminal methyl and ester carbonyl moieties adjacent to one another (Fig. 4A).^55^–^57^ X-ray crystallography and biophysical studies of CE (Fig. 4B) show that at lower temperatures, in the smectic phase, they also pack together in lamellar layers with their hydrocarbons, steroid nuclei, with terminal methyl and ester carbonyl moieties adjacent to one another and their side chains interdigitated.^17^,^58^ Adjacent terminal methyl moieties would be expected, especially with CE that contains branched terminal methyl groups. It is unknown how a mixture of WE and CE pack together, but it is reasonable that the minimal energy structures of CE and WE crystals alone are maintained in a mixture of the two. In the mixture, we speculate that the lipids would align to maximize hydrocarbon chain interactions just as they do for CE and WE alone (Fig. 5). The arrangement of molecules in Figure 5 allows for the interdigitation of CE side chains and maximizes the adjacent packing of the steroid nuclei and CE carbonyl moieties, just as they are in the crystalline state alone. The speculative model for the mixture of CE and WE (Fig. 5) has phospholipids with their hydrophilic head group facing the tear aqueous layer. Phospholipids do not interact with CE.^11^–^13^ It is speculative if the phospholipids in tears arise from debris^59^ or form a monolayer alone with other amphipathic molecules, such as OAHFA. Note that the carboxylate groups of the diester of OAHFA and the CE of OAHFA extend to the interface region where they may interact with water. For a 100-nm thick TFL layer, the motif structure of hydrophobic region and side chain-to-side chain region above the phospholipid monolayer in Figure 5 would stack about 26 times.

There are numerous limitations to the model in Figure 5. Proteins, especially mucin, are likely to associate with the tear lipid layer but are not shown in the model.^51^ Also, hydrocarbon chain kinks due to unsaturation as in Figure 4A are not shown in the model.

Cholesteryl palmitate had little effect on the phase-transition parameters of a mixture of stearyl palmitate and oleyloleate.^50^ However, cholesteryl behenate dramatically ordered and lowered the cooperativity of stearyl palmitate.^28^ From the model studies, it is difficult to predict the effect on structure of a major loss of CE with M_MGD_. As M_MGD_ is more ordered than M_n_,^50^ other factors such as saturation^44^,^54^ and/or proteins^49^,^51^ may be responsible for the higher order of M_MGD_. M_n_ order increases with age between 0 and 20 years of age. The CE/WE ratio increased with age between 0 and 20 years of age; thus, in addition to saturation,^44^,^54^ CE could contribute to the increase in order, with age between 0 and 20 years,^43^ as a cholesteryl behenate increased the order of WE.^28^ Experiments are underway to determine the contribution of CE to the order of meibum by separating meibum CE from WE and adding the moieties back together, as was done for saturated meibum.^44^,^54^

### Contribution of CE/WE to Function

The meibum content of CE alone is not likely to influence tear film (TF) stability given that a significant number of donors with a stable TF have lower meibum levels of CE, and a significant number of donors with MGD and an unstable TF have higher meibum levels of CE. Similarities in the rheology of feline, canine, and human meibum suggest that “comparable tear film dynamics have developed quite a robust mixture of meibomian lipids that is tolerable to significant variances in the ratios of some of its compounds and still establishes similar functionality.”^60^ In terms of function, such as for the cat and dog, because CE and WE are preferentially distributed within upper layers of the duplex tear film lipid (TFL) layer, they do not significantly contribute to the properties and lipid composition of the tear interface. Thus, large changes in the amount of CE in the TFL layer of humans can also be tolerated, resulting in comparable tear film dynamics. One should note that lipid hydrocarbon chain order driven by saturation does influence the surface properties of human meibum.^44^,^45^ There is no sex difference in TF stability (breakup times),^61^–^63^ which correlates with no sex difference in the CE of Mn in the current study. Correlations between TFL layer composition, structure and function may help elucidate the contribution of the TFLL to the signs and symptoms of dry eye and decreased tear film stability with age.

## Conclusions

Although M_MGD_ contains much less CE than M_n_, factors other than the CE content of meibum, such as the levels of saturation and/or proteins, may be responsible for the higher order of M_MGD_. In addition to saturation, CE could contribute to the increase in order of M_n_ between the ages of 0 and 20 years. Observed changes in the meibum content of CE alone is not likely to influence tear film stability.
